# Noninvasive biomarkers of gut barrier function identify two subtypes of patients suffering from diarrhoea predominant-IBS: a case-control study

**DOI:** 10.1186/s12876-018-0888-6

**Published:** 2018-11-06

**Authors:** Michele Linsalata, Giuseppe Riezzo, Benedetta D’Attoma, Caterina Clemente, Antonella Orlando, Francesco Russo

**Affiliations:** Laboratory of Nutritional Pathophysiology, National Institute of Gastroenterology, “S. de Bellis” Research Hospital, Via Turi 27, I-70013 Castellana Grotte, Bari, Italy

**Keywords:** Celiac disease, Diarrhoea-predominant irritable bowel syndrome, Gut barrier, Interleukins, Intestinal permeability, Lipopolysaccharide

## Abstract

**Background:**

Alterations of the small-intestinal permeability (s-IP) might play an essential role in both diarrhoea-predominant IBS (D-IBS) and celiac disease (CD) patients. Our aims were to analyse in D-IBS patients the symptom profile along with the levels of urinary sucrose (Su), lactulose (La), mannitol (Ma), and circulating biomarkers (zonulin, intestinal fatty acid binding protein - I-FABP, and diamine oxidase - DAO) of the gastrointestinal (GI) barrier function. The pro-inflammatory interleukins 6 and 8 (IL-6 and IL-8), the plasma values of lipopolysaccharide (LPS), and Toll-like receptor 4 (TLR-4) were also investigated. Besides, these biomarkers were compared with those in CD and healthy controls (HC). Finally, comparisons were performed between D-IBS patients with [D-IBS(+)] and without [D-IBS(−)] increased s-IP according to normal or altered La/Ma ratio.

**Methods:**

The study included 39 D-IBS patients, 32 CD patients, and 20 HC. GI permeability was assayed by high-performance liquid chromatography determination in the urine of Su and La/Ma ratio. ELISA kits assayed circulating concentrations of zonulin, I-FABP, DAO, IL-6, IL-8, LPS, and TLR-4. The Mann–Whitney or the Kruskal–Wallis with Dunn’s post-test was used to assess differences among the groups.

**Results:**

As for the La/Ma ratio, %Su, and I-FABP levels, D-IBS patients were significantly different from CD, but not HC. IL-6 levels were significantly higher in CD than HC, whereas IL-8 levels were significantly higher in both D-IBS and CD patients than HC. By opposite, LPS, and TLR-4 concentrations did not differ significantly among the groups. When D-IBS patients were categorised according to normal or altered s-IP, D-IBS(+) patients had %La, %Su, I-FABP, and DAO levels significantly higher than D-IBS(−) ones. The inflammatory parameters and markers of bacterial translocation (namely, IL-6 and LPS) were significantly higher in D-IBS(+) patients than D-IBS(−) ones.

**Conclusions:**

The present study suggests that two distinct D-IBS subtypes could be identified. The investigation of possible s-IP alterations (i.e., considering the La/Ma ratio) might be useful to assess better and categorise this heterogeneous D-IBS population.

**Trial registration:**

NCT01574209. Registered March 2012. First recruitment started in April 2012.

## Background

Irritable bowel syndrome (IBS) is a prevalent functional disorder in Italy, with percentages in the urban area (13.7%) double that in the rural area (5.9%) [1]. Although IBS still represents an underdiagnosed condition, it is one of the most important reasons for care-seeking within gastroenterology. The current IBS diagnosis is mainly based on the symptom criteria, stool characteristics [2], and specific questionnaires [3]. Additionally, as reported by the Bristol Stool Form Scale, the stool pattern features allow the categorisation of IBS subtypes in diarrhoea-predominant IBS (D-IBS), constipation-predominant IBS (C-IBS), mixed-type, and not classified [4].

Of note, the classic gastrointestinal (GI) symptom profile of the D-IBS subtype often mimics that of patients who have celiac disease (CD) (e.g., abdominal pain, bloating and diarrhoea) [5]. CD is a widespread autoimmune disorder characterised by chronic inflammation of the proximal small intestine, resulting in villous atrophy and malabsorption in genetically susceptible individuals after the ingestion of gluten [6]. Although clear key differences exist in their aetiology and treatment, the last years data in the literature has suggested that alterations in the intestinal barrier, mainly in the upper gut, might play an essential role in the development and perpetuation for both diseases [7, 8]. If on the one hand, gluten toxicity is a well-known cause of alterations in the small intestinal permeability (s-IP) of CD patients [9], on the other new insights into the aetiology of IBS have pointed out a role for low-grade inflammation in s-IP alterations of patients suffering from D-IBS [10]. Altered gut permeability can permit the passage of the luminal contents into the underlying tissues and thus into the bloodstream, resulting in both the activation of the immune response and the induction of gut inflammation. This permeability alteration is now considered the basis for the pathogenesis of many diseases, including IBS and CD. Therefore, the assessment of s-IP and the related molecular mechanisms may become an interesting parameter to consider in clinical practice for studying and treating these diseases [11]. However, no study has previously been performed aimed at comparing s-IP changes in these two diseases by applying the same methodologies.

Initial studies considered the use of single probes, such as ^51^Cr-EDTA, to assess the site of increased IP. This procedure, however, proved to be dependent on many non-mucosal factors, which not only reduced the sensitivity and specificity of the test but also posed a problem in the data interpretation. Moreover, the use of a radioactive substance such as ^51^Cr-EDTA exposes patients to radiation, thus putting a limit in its application in some patients (e.g. paediatric subjects, women of childbearing age, healthy subjects and patients requiring multiple permeability analyses) [12].

Nowadays, current methods for evaluating upper gut (gastric and small bowel) permeability use probes, such as small sugar molecules of different sizes. Among them, the most used are sucrose (Su), lactulose (La), and mannitol (Ma) [13]. Su, a disaccharide hydrolysed by the enzyme sucrase in the duodenum, has been proposed as a marker of gastric permeability [14]. La crosses the small intestinal barrier by paracellular passage if it is compromised, and it is considered a marker of tight junction (TJ) integrity. The smaller probe, Ma, crosses the epithelial barrier by transcellular passage, giving information on the whole epithelial absorptive area [15]. Since their urinary recovery is affected by several non-mucosal factors (e.g., gastric emptying, intestinal transit, and renal clearance), using a ratio rather than the single urinary recovery percentages overcomes such variations. For this reason, the La/Ma ratio in urine is considered a reliable parameter to evaluate the impairment of s-IP [14].

The intestinal barrier may be considered as a dynamic system also responding to humoral signals, and zonulin is one of the physiological modulators that regulate s-IP by changing TJ protein-protein interaction. It has been studied as a peripheral marker of IP in some diseases, and potential intestinal stimuli, such as gluten, can increase its secretion [16]. Intestinal barrier integrity is essential for s-IP. In this context, the intestinal fatty acid binding protein (I-FABP), a small cytosolic protein of 14 kDa specific to mature small bowel enterocytes, has proven to be a sensitive marker of damage to the intestinal epithelium, and its detection in the serum is suggestive for a breakdown of the enterocyte membrane [17]. Likewise, diamine oxidase (DAO), an intracellular enzyme with a high level of activity in the upper layer of intestinal villi, is considered another marker for the integrity of intestinal epithelium, whose serum levels increase in the case of damage and loss of barrier function [18]. Overall, few studies [19] have been conducted to evaluate these putative biomarkers of gut integrity in patients suffering from D-IBS.

In order to improve our knowledge about s-IP and the integrity of the GI barrier as well as their implications for D-IBS pathophysiology, the aims of this study were to (a) analyse the symptom profile using a validated questionnaire such as the Gastrointestinal Symptom Rating Scale (GSRS) [20] in D-IBS patients and compare it with those recorded in CD patients and healthy controls (HC); (b) evaluate the levels of urinary (La, Ma, and Su) and circulating (zonulin, I-FABP, and DAO) biomarkers of function and integrity of the GI barrier along with the pro-inflammatory interleukins 6 and 8 (IL-6 and IL-8), the plasma values of lipopolysaccharide (LPS), and Toll-like receptor 4 (TLR-4) in D-IBS patients. Comparisons with the results obtained from CD patients and HC subjects were then performed; and (c) compare GI symptoms and the above-mentioned urinary and circulating markers in D-IBS patients with increased s-IP, as diagnosed by the La/Ma ratio, [D-IBS(+)], with those in D-IBS patients with normal s-IP, [D-IBS(−)].

## Methods

### Study participants

Patients suffering from diarrhoea-predominant IBS according to Rome III criteria, were recruited in this prospective case-control study from among the outpatients of the National Institute of Gastroenterology, “S. de Bellis” Research Hospital, Castellana Grotte, Italy.

The inclusion criteria were: (*a*) age more than 18 years; (*b*) a symptom profile resembling D-IBS with a stool pattern, as described according to Schmulson et al. [21]; (*c*) active symptoms for at least 2 weeks; (*d*) a minimum average of 3.0 on the seven-point Likert scale of the GSRS composite symptom score [20]; (*e*) a diet without any restrictions on eating and drinking (in particular, no previous period of gluten free diet (GFD) before examination); (*f*) as gluten-sensitive diarrhoea without CD is a clinical entity that has been observed in IBS patients positive for HLA-DQ2 or HLA-DQ8 [22], only the HLA-DQ2/HLADQ8-negative/negative D-IBS patients were considered for this study; (*g*) age, body mass index (BMI), anxiety or depression, smoking, alcohol intake and use of medication were accurately checked in order to obtain a group of D-IBS patients as homogeneous as possible.

All the patients underwent a physical examination, whole blood count, liver function tests, stool routine, faecal occult blood test, stool culture, stool examination for parasites, C-reactive protein, thyroid function test, gastroscopy, and colonoscopy in order to exclude patients with organic diseases. As concerns the female patients, to avoid any possible interference and contamination of the urine samples with blood, the urinary and blood samples were obtained within 10 days of the onset of the most recent menstrual cycle (follicular phase).

The diagnosis of CD was performed following the international guidelines and published data [23]. Serologic testing, with a combination of tissue transglutaminase (tTG) and anti-endomysium antibodies (EMA), was used. For recruitment in the study as CD patients, the diagnosis of CD had to be confirmed with a duodenal biopsy sample according to the modified Marsh–Oberhuber criteria (grades 3b–3c) [24].

Exclusion criteria included: post-infectious IBS, hepatic, renal or cardiovascular disease, constipation, metabolic and endocrine disorders, fever, intense physical activity, previous abdominal surgery, history of malignancy, secondary causes of intestinal atrophy, pregnancy, lactose intolerance or giardiasis. Besides, patients did not have to consume medication for the treatment of IBS for 2 weeks before evaluation, antibiotic therapy or probiotic agents, and other drugs known to cause abdominal pain.

The reasons for study discontinuation were recorded in the case report form and could include: death, adverse event (specified), ineligibility to continue the study, lost to follow-up, withdrew consent, and other (including the administrative closure of trial).

Healthy individuals were enrolled from among the administrative staff of our Institute as healthy controls (HC). They denied having metabolic, endocrine, or immunological diseases, dyspepsia, or other GI diseases and did not take any medication. Information on the health status of participants was obtained by an interview on the current diet, lifestyle, medical history, and a physical examination. As criteria for admission, EMA and tTG had to be negative. Besides, metabolic parameters (blood glucose, HbA1c, lipid profile, body weight, and blood pressure) had to be within the normal range of values. The absence of major psychiatric disorders, cancer, and pregnancy were also inclusion criteria. All the women, either patients or controls, were examined during the follicular phase of the menstrual cycle.

All the participants belonging to the three distinct groups (D-IBS, CD, and HC) were subjected to all the scheduled analyses. The CD patients were considered as positive controls. The HC subjects were enrolled as negative controls (study 1). After the D-IBS patients were separated into the D-IBS(+) and D-IBS(−) groups, according to whether s-IP was altered or not at the La/Ma ratio, the clinical characteristics and the urinary and circulating parameters of the two D-IBS subgroups, were evaluated (study 2).

All the subjects were compliant and were willing to participate in the study. Written informed consent was obtained from all the patients and healthy participants for blood testing and clinical data collection. This study was approved by the Institutional Ethics Committee of IRCCS Ospedale Oncologico di Bari - Istituto Tumori Giovanni Paolo II, Bari, Italy, DDG reg. 1227/2013, and it was part of registered research on http://www.clinicaltrials.gov (reg. Number: NCT01574209).

### Symptom assessment

Patients were evaluated with the GSRS, a validated questionnaire for GI symptoms [20]. GSRS utilizes a seven-level Likert scale (1–7), depending on intensity and frequency of GI symptoms experienced during the previous week. A higher score indicates mainly inconvenient symptoms. Combination scores among the questions can assess the following five domains: “*reflux syndrome*” (halitosis, heartburn, dysphagia and acid regurgitation: max. Score: 28), “*abdominal pain*” (pain referred as epigastric, colic, continuous or indefinite pain, gastric hunger pains and nausea: max. Score: 42), “*indigestion syndrome*” (postprandial fullness, early satiety, borborygmi, bloating, eructation/belching and increased flatus, max. Score: 42), “*diarrhoea syndrome*” (increased frequency of evacuation, loose stools and urgent need to defecate, max. Score: 21), and “*constipation syndrome”* (reduced frequency of evacuation, hard stools and feeling of incomplete evacuation, max. Score: 21). In the case of D-IBS patients, *“abdominal pain”, “indigestion syndrome”,* and *“diarrhoea syndrome”* were taken into account. The stool consistency was investigated using the Bristol stool form chart [4].

### Serological assay

All the analytical measurements were performed at the time of enrolment using blind-coded samples (no name or personal identifiers). Peripheral venous blood samples were obtained from participants in the study in the fasting state at least 12 h after the last meal.

After allowing to clot for at least 30 min, the samples were centrifuged at 1600 g for 15 min.

The serum samples were stored at − 80 °C until the assay and tested for immunoglobulin A (IgA) anti-EMA by the indirect immunofluorescence technique using sections of Monkey oesophagus as a substrate and anti-human IgA fluorescein as a conjugate (NOVA Lite Monkey Esophagus IFA it/slides; Inova Diagnostic Inc., San Diego, California, USA) following the instructions of the manufacturer. Slides were examined under a fluorescence microscope to identify the presence of autoantibody. Endomysial positive control, derived from human serum, and negative control, entirely negative for all autoantibodies, were included in every run.

The analysis of IgA anti-tTG was carried out using an enzyme immunoassay (EliACelikey IgA Well; Thermo Fisher Scientific, Waltham, Massachusetts, USA) and performed on the fully automated system (Phadia 250; Phadia GmbH, Freiburg, Germany). All samples were double analysed in a blinded manner, with the addition of positive and negative controls for each analysis run.

Serum levels of I-FABP in peripheral blood were evaluated by enzyme-link immunosorbent assay (ELISA) using a specific anti-human I-FABP antibody (Thermo Fisher Scientific, Waltham, Massachusetts, USA). DAO levels were determined by a commercially available ELISA Kit (Cloud-Clone Corp. Houston, USA). Zonulin was assayed using the specific ELISA kit (Immunodiagnostik AG, Bensheim, Germany).

Plasma levels of IL-6, IL-8, LPS, and TLR-4 were measured in duplicate using commercially available sandwich enzyme-linked immunosorbent assay kits (Human IL-6 ELISA and Human IL-8 ELISA, BD Biosciences, Milan, Italy; Lipopolysaccharide (LPS) ELISA kit Cloud-Clone Corp., Katy, TX, USA; Human Toll-Like Receptor 4 (TLR-4) ELISA kit Cloud-Clone Corp., Katy, TX, USA).

### Sugar absorption tests

For the evaluation of GI permeability, all the participants fasted overnight. In order to check for the possible presence of endogenous sugars, a pretest urine was collected in our laboratory. Then subjects drank a sugar test solution containing 10 g of lactulose, 5 g of mannitol and 40 g of sucrose in a volume of 100 ml. Urine samples from control and patient subjects were collected up to 5 h after administration. A l-ml volume of 20% (*w*/*v*) chlorohexidine was added to each collection as a preservative regardless of the final total volumes. The total urine volumes from individuals were measured and recorded. After thoroughly mixing, a portion of 2 ml was taken and stored at − 80 °C until analysed.

The detection and measurement of the three sugar probes, Su, La, and Ma in urine were performed by chromatographic analysis as described previously by our group [25]. Briefly, high-performance anion exchange chromatography coupled with pulsed amperometric detection was performed on a Dionex Model ICS-5000 with a gold working electrode, and a 25 μl peek sample loop (Dionex Corp., Sunnyvale, California, USA).

The carbohydrate separation was performed using a Carbopac PA-10 pellicular anion-exchange resin connected to a Carbopac PA-10 guard column (Thermofisher Scientific, Waltham, Massachusetts, USA) at 30 °C. The samples were eluted with 50 mmol/l NaOH at a flow rate of 1 ml/min. The percentage of ingested Su (%Su) together with those of La (%La) and Ma (%Ma) in urine were evaluated, and the La/Ma ratio was calculated for each sample.

Patients with a La/Ma ratio lower than 0.035 were considered as D-IBS(−); patients with a value equal to or higher than 0.035 were considered D-IBS(+). This cut-off value (mean + 2SD) derived from our previous study performed on a large group of healthy subjects [26].

### Statistical analysis

All results are expressed as mean ± SEM unless otherwise specified. Data analysis concerned the comparisons of symptom profile, s-IP, markers of barrier function, and markers of inflammation among HC, D-IBS and CD patients (Study 1) and the comparison of the same variables when D-IBS patients were categorized as D-IBS(−) and D-IBS(+) (Study 2). Non-parametric tests were performed to avoid violation of the assumption of normal distribution. The Mann–Whitney or the Kruskal–Wallis with Dunn’s post-test was used to assess differences among two or more the groups, respectively. Pearson’s correlation coefficient measures the statistical relationship, or association, between two continuous variables. It is known as the best method of measuring the association between variables of interest because it is based on the method of covariance. The correlation coefficient r was calculated among the urinary and circulating IP biomarkers and the inflammatory parameters. All the differences were considered significant at a 5% level. A specific statistical package for exact nonparametric inference (2005 Stata Statistical Software Release 9; Stata Corp., College Station, Texas, USA) was used.

## Results

### Study 1. Comparisons among D-IBS patients, celiac disease patients, and healthy controls

**Fig. 1:**
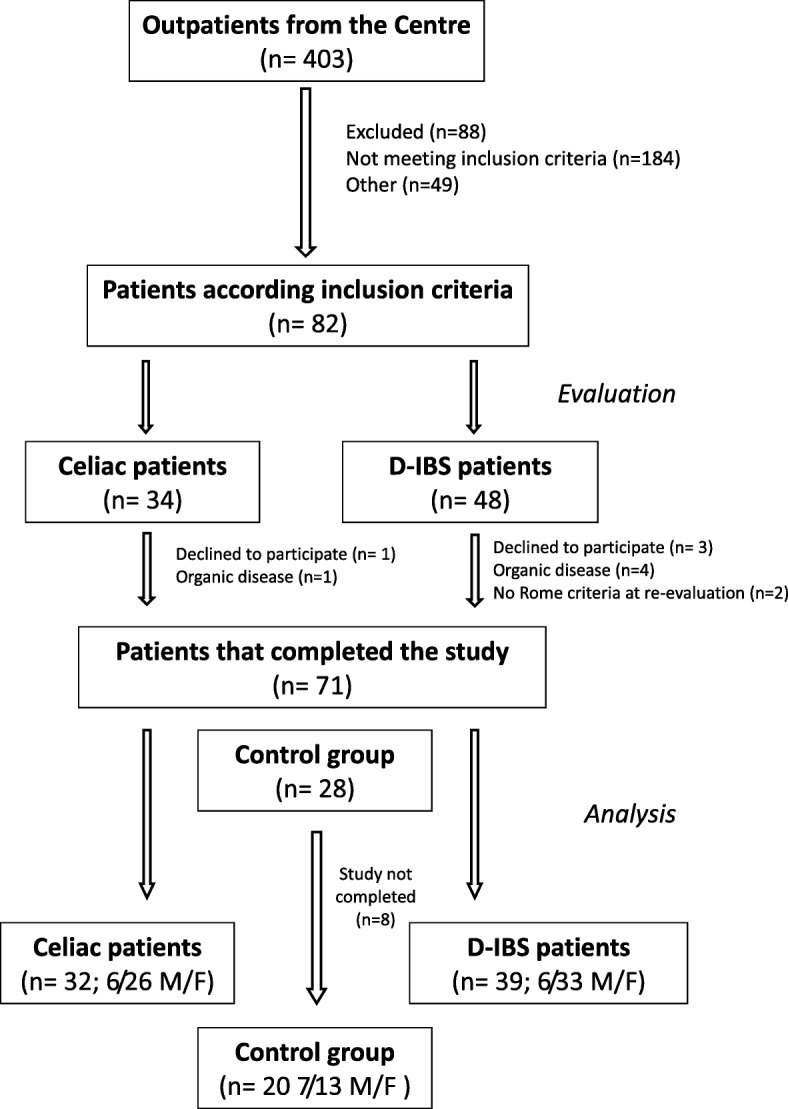
The flow of participants through the study D-IBS = diarrhoea-predominant IBS

Figure 1 shows the flow of participants through the study. Four hundred and three participants were included. Of these, 184 patients did not fulfil the inclusion criteria; 88 patients were excluded due to refusal to undergo endoscopy; 49 patients did not enter the study for other reasons. Thus, 82 patients were considered for the study: 34 adult celiac patients with diarrhoea as the prevalent GI symptom and 48 D-IBS patients. One CD patients declined to participate, and one underwent major surgery. Three D-IBS patients refused to participate, four suffered from organic diseases, and two did not meet Rome III criteria when they were re-evaluated. The HC group comprised 28 subjects, but 8 of them did not complete the study. As a result, 20 HC subjects were analysed.

Table 1 describes the anthropometric characteristics and clinical data of the HC, D-IBS, and CD patients.

**Table 1:** Anthropometric and clinical data (GSRS items) of HC, D-IBS, and CD patients

	HC	D-IBS	CD
Anthropometric parameters			
Sex	7/13 (M/F)	6/33 (M/F)	6/26 (M/F)
Age (yrs.)	39.7 ± 7.2^a^	40.05 ± 12.2^a^	35.9 ± 3.71^a^
BMI	23.8 ± 2.9^a^	23.9 ± 3.3^a^	22.39 ± 3.65^a^
GSRS single items			
Nausea/vomiting	1.0 (1–1)^a^	1.0 (1–6)^b^	1.0 (1–6)^b^
Abdominal pain (colic pain)	1.0 (1–1)^a^	3.0 (1–7)^b^	2.0 (1–7)^b^
Gastric hunger pain	1.0 (1–1)^a^	2.0 (1–7)^b^	2.0 (1–7)^b^
Abdominal distension	1.0 (1–1)^a^	5.0 (1–7)^b^	5.0 (1–7)^b^
Burping	1.0 (1–1)^a^	2.0 (1–7)^b^	1.0 (1–7)^b^
Borborygmi	1.0 (1–1)^a^	3.0 (1–7)^b^	3.0 (1–7)^b^
Flatulence	1.0 (1–1)^a^	4.0 (1–7)^b^	4.0 (1–7)^b^
Increased passage of stools	1.0 (1–1)^a^	1.0 (1–7)^b^	2.0 (1–7)^b^
Bristol score	3.0 (3–4)^a^	4.0 (3–7)^b^	4.0 (2–7)^b^
Urgent bowel movement	1.0 (1–1)^a^	3.0 (1–7)^b^	3.0 (1–7)^b^
Feeling of incomplete defecation	1.0 (1–1)^a^	3.0 (1–7)^b^	2.5 (1–5)^b^
GSRS combination scores			
Abdominal pain	6.0 (6–6)^a^	14 (6–25)^b^	13.5 (6–29)^b^
Indigestion syndrome	6.0 (6–6)^a^	19.0 (7–38)^b^	20.0 (7–42)^b^
syndrome	3.0 (3–3)^a^	5.0 (3–21)^b^	6.5 (3–19)^b^

*HC* healthy controls, *D-IBS* diarrhoea-predominant IBS patients, *CD* celiac disease patients. Continuous data are expressed as Mean ± SD, and discrete data are expressed as Median and range. All data were analysed by Kruskal–Wallis test with Dunn’s post-test. Different superscripts differ significantly (*p* < 0.05)

Anthropometric data were not significantly different among the groups. GSRS questionnaire items, such as single and combination items, were similar between D-IBS and CD patients. As expected, the GSRS scores recorded in both patients groups were significantly (*p* < 0.05) different from that in HC subjects.

**Fig. 2:**
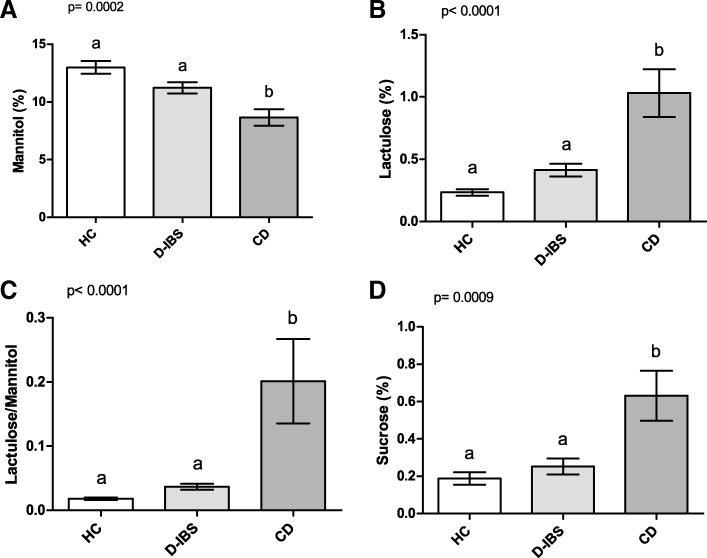
%Ma, %La, La/Ma, and %Su in HC, D-IBS, and CD patients. **A **%Ma = Percentage of ingested mannitol recovered in urine. **B **%La = Percentage of ingested lactulose recovered in urine; **C** La/Ma = lactulose to mannitol ratio;  **D** %Su = Percentage of ingested sucrose recovered in urine. HC = Healthy controls. D-IBS = diarrhoea-predominant IBS. CD = celiac disease. Data are expressed as Mean ± SEM and analysed by Kruskal-Wallis test with Dunn’s Multiple Comparison Test. Means sharing the same superscript are not significantly different from each other (*p* < 0.05, Dunn’s test)

All the HC, D-IBS, and CD subjects underwent IP testing (Fig. 2). As for Ma (Fig. 2A), significant differences were present among the groups (*p* = 0.0002). D-IBS patients and HC subjects showed significantly higher %Ma compared to CD patients (*p* < 0.05 and *p* < 0.01, respectively) at the post hoc test. Significant differences were also present among the three groups (*p* < 0.0001) for %La. D-IBS patients and HC subjects showed significantly (*p* < 0.001) lower %La compared to CD patients at the post hoc test (Fig. 2B). Consequently, the La/Ma ratio differed significantly among the groups (*p* < 0.0001), and D-IBS patients and HC subjects had significantly (*p* < 0.001) lower ratio values than CD patients (Fig. 2C). Finally, %Su also differed significantly (*p* = 0.0009) among the groups, and both D-IBS and HC subjects had significantly (*p* < 0.01) lower values than CD patients (Fig. 2D).

**Fig. 3:**
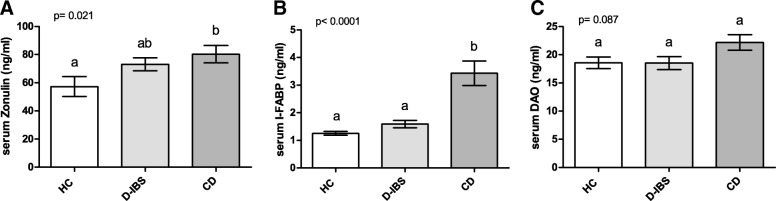
Serum Zonulin, I-FABP, and DAO levels in HC, D-IBS, and CD patients. **A **Zonulin; **B **I-FABP = Intestinal fatty acid binding protein; **C** DAO = diamine oxidase. HC = Healthy controls. D-IBS = diarrhoea-predominant IBS. CD = celiac disease. Data are expressed as Mean ± SEM and analysed by Kruskal-Wallis test with Dunn’s Multiple Comparison Test. Means sharing the same superscript are not significantly different from each other (*p* < 0.05, Dunn’s test)

Figure 3 shows the zonulin, I-FABP, and DAO levels in the serum of patients and controls. Zonulin levels differed significantly (*p* = 0.021) among the HC, D-IBS, and CD groups. The latter group had significantly (*p* < 0.05) higher circulating levels than HC subjects but not D-IBS patients (Fig. 3A). I-FABP concentrations were significantly (*p* < 0.0001) different among the groups, and both D-IBS patients and HC subjects had significantly (*p* < 0.001) lower values than CD patients (Fig. 3B). On the contrary, the DAO levels were not significantly (*p* = 0.087) different among the groups (Fig. 3C).

**Fig. 4:**
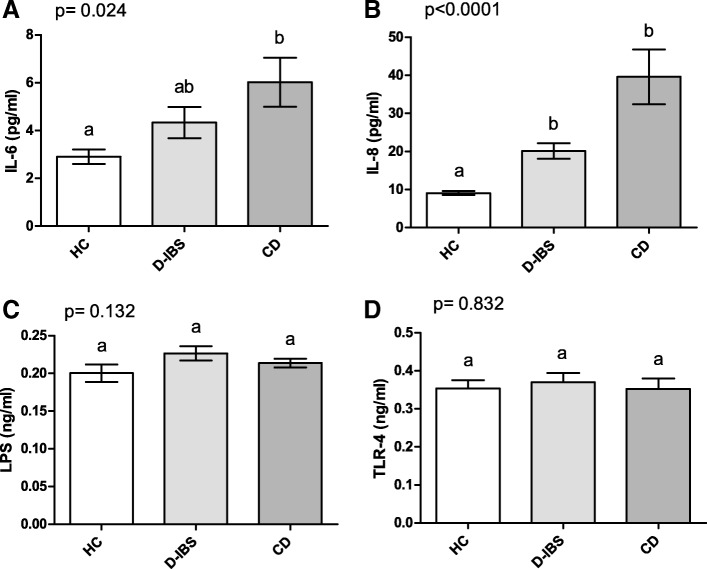
Plasma concentrations of IL-6, IL-8, LPS, and TLR-4 in HC, D-IBS, and CD patients. **A** IL-6 = Interleukin-6;  **B** IL-8 = Interleukin-8; **C** LPS = Lipopolysaccharide; **D** TLR-4 = Toll-like receptor 4. HC = Healthy controls. D-IBS = diarrhoea-predominant IBS. CD = celiac disease. Data are expressed as Mean ± SEM and analysed by Kruskal-Wallis test with Dunn’s Multiple Comparison Test. Means sharing the same superscript are not significantly different from each other (*p* < 0.05, Dunn’s test)

Figure 4 reports the values of circulating IL-6 and IL-8 along with the circulating concentrations of LPS and TLR-4. As concerns cytokines, the values of IL-6 were significantly (*p* = 0.024) different among the three groups, and CD patients showed significantly (*p* < 0.05) higher levels than HC subjects at the post hoc test (Fig. 4A). IL-8 was also significantly (*p* < 0.0001) different among the groups and not only CD but also D-IBS patients had significantly (*p* < 0.001) higher concentrations than HC subjects (Fig. 4B). Lastly, both the plasma LPS and TLR-4 levels did not differ significantly among the groups (*p* = 0.132 and *p* = 0.832, respectively) (Fig. 4C and D).

Table 2 reports the Pearson correlation coefficients (r) between the urinary and circulating IP biomarkers and the inflammatory parameters in the whole population studied. Significant positive correlations (*p* < 0.0001) were present between I-FABP and IL-8, I-FABP and %La, I-FABP and La/Ma ratio. The La/Ma ratio positively and significantly (*p* < 0.0001) also correlated with IL-8. A significant negative correlation (*p* < 0.001) was present between %Ma and IL-8. Of note, %Su, a marker of gastroduodenal permeability, significantly (*p* < 0.0001) correlated with %La, La/Ma ratio, and I-FABP. No correlations between GSRS symptoms and urinary sugars as well as GSRS symptoms and each serum biomarker were recorded. The length of time since the IBS started and the symptoms and permeability markers did not correlate (data not shown).

**Table 2:** The Pearson correlation coefficients (r) among the inflammatory parameters and the urinary and circulating intestinal permeability biomarkers in the whole population studied (n. 91 cases)

	%Ma	%La	La/Ma	%Su	Zonulin	IFAB-P	DAO	IL-6	IL-8	LPS	TLR-4
%Ma	1										
%La	−0.13	1									
La/Ma	−0.41****	0.93****	1								
%Su	0.09	0.67****	0.58****	1							
Zonulin	−0.22*	0.12	0.17	− 0.12	1						
IFAB-P	−0.26*	0.61****	0.67****	0.47****	0.10	1					
DAO	0.04	0.23*	0.27**	0.17	0.10	0.24*	1				
IL-6	0.05	0.32**	0.32**	0.18	0.24*	0.33**	0.23*	1			
IL-8	−0.36***	0.33**	0.42****	0.16	0.31**	0.45****	0.22*	0.31**	1		
LPS	0.05	0.15	0.14	0.06	0.15	0.07	−0.02	0.27*	0.18	1	
TLR-4	0.16	0.14	0.04	0.7	0.14	0.07	−0.15	0.09	0.07	0.26*	1

*%La* percentage of ingested lactulose recovered in the urine, *%Ma* percentage of the ingested mannitol recovered in urine, *La/Ma* lactulose to mannitol ratio, *%Su* percentage of ingested sucrose recovered in the urine, *I-FABP* Intestinal fatty acid binding protein, *DAO* diamine oxidase

^*^p < 0.05

^**^p < 0.01

^***^p < 0.001

^****^p < 0.0001

### Study 2. Differences between D-IBS patients with normal and altered s-IP

When the patients were categorised according to normal or altered s-IP, 28 out 32 CD patients (87.5%) and 18 out 39 D-IBS patients (46.2%) had a La/Ma ratio equal to or higher than 0.035 [D-IBS(+)]. All the controls and 21 out of 39 D-IBS patients (53.8%) had a La/Ma ratio lower than 0.035 [D-IBS(−)].

Table 3 describes the anthropometric and clinical data of D-IBS(+) and D-IBS(−) patients. As for the GSRS single and combination scores, the two groups showed no significant difference for both the GSRS single items and combination scores.

**Table 3:** Anthropometric and clinical data (GSRS items) of D-IBS patients according to normal D-IBS(−) or altered D-IBS(+) small intestinal permeability

	D-IBS(−)	D-IBS(+)	
Anthropometric parameters			
Sex	2/19 (M/F)	4/14 (M/F)	
Age (yrs.)	39.89 ± 11.25	40.19 ± 13.24	ns
BMI	24.48 ± 2.69	23.44 ± 3.76	ns
GSRS single items			*p*
Nausea/vomiting	2.0 (1–6)	1.0 (1–4)	ns
Abdominal pain (colic pain)	2.0 (1–6)	4.5 (1–7)	ns
Gastric hunger pain	5.0 (1–7)	2.0 (1–6)	ns
Abdominal distension	5.0 (1–7)	5.0 (1–7)	ns
Burping	1.0 (1–7)	3.0 (1–7)	ns
Borborygmi	3.0 (1–7)	2.5 (1–7)	ns
Flatulence	5.0 (1–7)	3.0 (1–6)	ns
Increased passage of stools	1.0 (1–3)	1.0 (1–7)	ns
Bristol score	5.0 (3–6)	4.0 (3–7)	ns
Urgent bowel movement	3.0 (1–7)	3.0 (1–7)	ns
Feeling of incomplete defecation	4.0 (1–7)	3.0 (1–7)	ns
GSRS combination scores			
Abdominal pain	13.0 (8–25)	14.0 (6–25)	ns
Indigestion syndrome	19.0 (12–36)	19.5 (7–39)	ns
Diarrhea syndrome	8.0 (3–21)	6.0 (3–13)	ns

*D-IBS* diarrhoea-predominant IBS, *D-IBS* patients with a lactulose to mannitol ratio lower than 0.035 were considered D-IBS(−); patients with a ratio value equal to or higher than 0.035 as D-IBS(+). Continuous data are expressed as Mean ± SD, and discrete data are expressed as Median and range. Data were analysed by Mann Whitney test

**Fig. 5:**
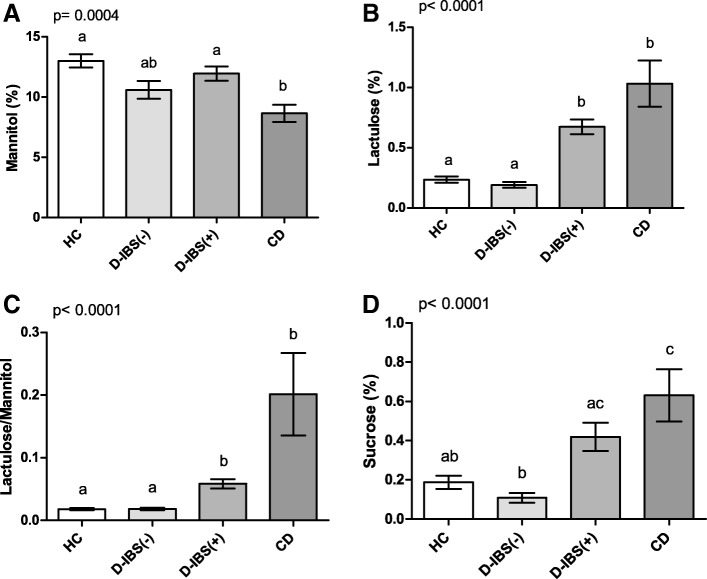
Urinary markers of gastrointestinal barrier function in HC, D-IBS(−),D-IBS(+) patients, and CD patients HC = healthy controls. D-IBS = diarrhoea-predominant IBS. D-IBS patients with a Lactulose to Mannitol ratio lower than 0.035 were considered D-IBS(−). D-IBS patients with a ratio value equal to or higher than 0.035 were considered as D-IBS(+). CD = celiac disease. Urinary parameters of gastrointestinal permeability are expressed as percentages of ingested sugars recovered in urine: **A** mannitol (%Ma), **B** lactulose (%La), **C** the La/Ma ratio, and **D** sucrose (%Su). Data are expressed as Mean ± SEM and analysed by Kruskal-Wallis test with Dunn’s Multiple Comparison Test. Means sharing the same superscript are not significantly different from each other (*p* < 0.05, Dunn’s test)

Figure 5 reports the urinary markers of GI barrier function in HC, D-IBS(−), D-IBS(+), and CD patients.

**Fig. 6:**
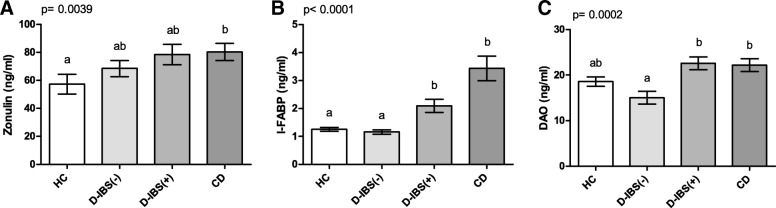
Circulating markers of intestinal barrier function in HC, D-IBS(−), D-IBS(+) patients, and CD patients. HC = healthy controls. D-IBS = diarrhoea-predominant IBS. D-IBS patients with a Lactulose to Mannitol ratio lower than 0.035 were considered D-IBS(−). D-IBS patients with a ratio value equal to or higher than 0.035 were considered as D-IBS(+). CD = celiac disease. Circulating parameters of gastrointestinal permeability are expressed as: **A** Zonulin; **B** I-FABP = Intestinal fatty acid binding protein; **C** DAO = diamine oxidase. Data are expressed as Mean ± SEM and analysed by Kruskal-Wallis test with Dunn’s Multiple Comparison Test. Means sharing the same superscript are not significantly different from each other (*p* < 0.05, Dunn’s test)

%Ma differed significantly (*p* = 0.0004) among the four groups, and both D-IBS(+) patients and HC subjects had significantly higher percentages of sugar excretion than CD patients (*p* < 0.05 and *p* < 0.001, respectively) (Fig. 5A). As concerns %La, significant (*p* < 0.0001) differences were present among the groups. At the post hoc test, both D-IBS(+) and CD patients had significantly (*p* < 0.001) higher %La than D-IBS(−) patients and HC subjects (Fig. 5B). This evidence indicates a failure in the paracellular permeability of the former two groups. Consequently, the La/Ma ratio differed significantly (*p* < 0.0001) among the groups, and both D-IBS(+) and CD patients had significantly (*p* < 0.001) higher ratio values than D-IBS(−) patients and HC subjects (Fig. 5C). A significant difference was also found in %Su among the groups (*p* < 0.0001), and D-IBS(+) and CD patients had significantly (*p* < 0.001) higher concentrations compared to D-IBS(−) patients. Besides, %Su in CD patients was significantly higher than that in HC subjects (*p* < 0.05) (Fig. 5D). When the serum markers of barrier function were compared (Fig. 6), significant (*p* = 0.0039) differences in the zonulin levels were present among the groups, and the CD patients showed significantly (*p* < 0.05) higher levels than HC subjects at the post hoc test (Fig. 6A). The circulating levels of I-FABP were significantly (*p* < 0.0001) different among the four groups, and both D-IBS(+) and CD patients showed significantly (*p* < 0.01) higher levels than D-IBS(−) and HC subjects (Fig. 6B). Finally, DAO concentrations differed significantly (*p* = 0.0002) among the four groups. At the post hoc test, both D-IBS(+) and CD patients showed significantly (*p* < 0.001) higher circulating levels compared to D-IBS(−) patients (Fig. 6C).

**Fig. 7:**
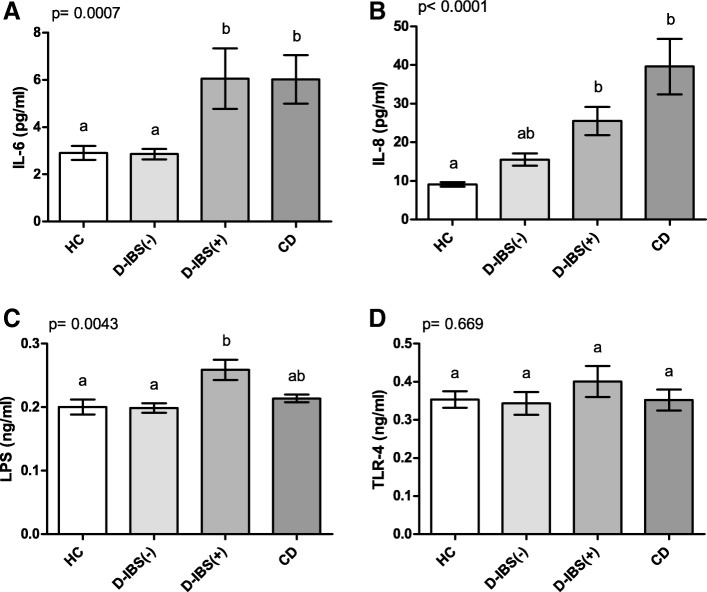
Circulating levels of IL-6, IL-8, LPS, and TLR-4 in HC, D-IBS(−), D-IBS(+) patients, and CD patients. HC = healthy controls. D-IBS = diarrhoea-predominant IBS. D-IBS patients with a Lactulose to Mannitol ratio lower than 0.035 were considered D-IBS(−). D-IBS patients with a ratio value equal to or higher than 0.035 were considered as D-IBS(+). CD = celiac disease. **A** IL-6 = Interleukin-6; **B** IL-8 = Interleukin-8; **C** LPS = Lipopolysaccharide; **D** TLR-4 = Toll-like receptor 4. Data are expressed as Mean ± SEM and analysed by Kruskal-Wallis test with Dunn’s Multiple Comparison Test. Means sharing the same superscript are not significantly different from each other (*p* < 0.05, Dunn’s test)

Figure 7 reports the circulating levels of IL-6, IL-8, LPS, and TLR-4 in HC, D-IBS(−), D-IBS(+), and CD patients. Significant differences (*p* = 0.0007) were found in the IL-6 levels among the groups. D-IBS(+) and CD patients had significantly (*p* < 0.05) higher concentrations than D-IBS(−) patients and HC subjects (Fig. 7A). As concerns IL-8, D-IBS(+) and CD patients had significantly (*p* < 0.01) higher circulating levels compared to HC but not D-IBS(−) patients (Fig. 7B). LPS concentrations were significantly (*p* = 0.0043) different among the groups (Fig. 7C). At the post hoc test, D-IBS(+) patients showed the highest LPS levels reaching a significant (*p* < 0.01) difference compared to both D-IBS(−) patients and HC subjects. Finally, TLR-4 did not show significant differences among the groups (*p* = 0.669) (Fig. 7D).

## Discussion

Although contrasting and often unclear, many pieces of evidence suggest that alterations of the intestinal barrier may play an essential role in the pathogenesis of IBS, particularly in its diarrhoea-predominant variant [27]. A defective epithelial barrier function, which can be measured as increased gut permeability, could facilitate passage of luminal antigens and lead to a mucosal immune response. The identification of increased s-IP in patients with D-IBS may become relevant from a therapeutic perspective [28].

In the present study, we firstly focused on the evaluation of the D-IBS symptom profile, the urinary and serum markers of the GI epithelium function and inflammatory parameters. Then, we compared the obtained findings with those from CD patients and healthy subjects.

In spite of their well-known different aetiology, D-IBS and CD patients were not different in their symptomatology as concerns bowel habits and abdominal symptoms. This finding may account for the limited ability of questionnaires in efficiently discriminating between patients, due to the frequent overlapping symptom profiles, as also described in other GI diseases [29]. Moreover, it suggests the need for new strategies for IBS classification and diagnosis, with the use of new bio-humoral markers that can help clinicians in its management. In this context, our cohort of D-IBS patients showed significantly lower levels of La/Ma ratio and I-FABP levels compared to CD patients. This finding may be imputable to the minor altered epithelium permeability as well as the less evident damage in the intestinal integrity in D-IBS patients compared to patients suffering from systemic autoimmune diseases (e.g., celiac disease, considered here as a putative positive control). Evident alterations in GI permeability characterise celiac disease, and as expected, the excretions of Su, La, and Ma as well as the La/Ma ratio were significantly different from those in the HC group. These data are in full agreement with the previously reported alterations in CD patients for gastric and s-IP, the latter characterised by both modifications of TJ and decreased mucosal absorptive surface [30]. In line with the results obtained with urinary markers, serum levels of I-FABP were higher in CD patients compared to both D-IBS patients and HC subjects. As a further demonstration of the close relationship between altered s-IP and mucosal damage, I-FABP strongly and positively correlated with either %La or La/Ma ratio. Finally, zonulin levels were also significantly higher in CD patients than in HC subjects, in full accordance with published data [31].

As a whole group, D-IBS patients did not significantly differ from HC subjects for the secretion of the urinary markers of GI permeability. Unfortunately, there is no uniformity of data on this issue. Some papers already reported no alterations in the GI permeability of IBS patients [32–34], whereas other studies described impairment in the GI barrier function when D-IBS patients were evaluated [28, 35, 36]. These divergent findings could be due to either the different methods applied or the different criteria adopted for patient selection and diagnosis. Additionally, recent studies showed that epithelial barrier dysfunction is localised only to the small intestine in D-IBS patients, and no differences between IBS patients and controls in colonic permeability has been found [37]. However, the involvement of changes in colonic permeability in IBS is still under debate, since other studies reported increased colonic permeability [32].

Our results on the urinary markers of GI permeability were confirmed by those of zonulin and the serum integrity markers of the epithelium. Firstly, there were no significant differences between D-IBS patients and HC subjects in zonulin levels. These findings are congruent with a recent study by Ohlsson et al. [38]. In that study, subjects with a history of functional GI symptoms (IBS and also functional dyspepsia) had the same zonulin levels as those without symptoms. This result may not be unexpected if we consider that the pathophysiology of D-IBS is still under investigation and several agents might play a role in its aetiology. Although zonulin regulates TJ and IP permeability, a plethora of different proteins are known to participate in this regulation. So, in agreement with Ohlsson et al. [38], attention must be paid before considering only serum zonulin as a biomarker of s-IP. Secondly, as concerns I-FABP levels, following our results, the only study available in the literature [33] showed no significant differences between HC and IBS patients, either before or after NSAID consumption, indicating the absence of damage to the intestinal epithelium in this functional GI disease. We also found no significant increase in the DAO serum levels of D-IBS patient compared to HC subjects, although this evidence is not in agreement with available data [19]. However, given the limited experience in the clinical use of serum DAO by our and other groups, it needs to be verified by further investigation. Of note, the analysis of the pro-inflammatory IL-8 and IL-6 showed higher levels in D-IBS patients compared to HC subjects, although statistical significance was present only for the former cytokine. This finding supports the notion of low-grade inflammation in this disease [10]. Besides, significant correlations between these pro-inflammatory cytokines and the circulating and urinary markers of GI permeability were found in the whole population studied. These pieces of evidence suggest the close relationship between the changes in barrier function and inflammatory processes. Under physiological conditions, the GI epithelium provides an effective barrier between the internal and external environment, protecting the body from potentially harmful luminal substances such as bacterial products, digestive enzymes, and antigens. The loss of integrity of the GI barrier is accompanied by an increase in epithelial permeability, reflecting a state in which luminal substances can permeate the barrier and enter the systemic circulation, where they may contribute to a systemic inflammatory response and organ dysfunction [12].

Another aim of the present work was to evaluate D-IBS patients according to the presence of normal or increased s-IP. Categorisation was performed to investigate whether this alteration could affect the symptom profile as well as biomarkers of gut barrier function and inflammation. In our study, 46% of D-IBS patients showed increased s-IP as diagnosed by the La/Ma ratio, in spite of the absence of significant differences in the symptom profile.

To date, differences in the symptomatology between D-IBS patients with normal or increased s-IP have not been investigated in-depth. In 2009, Zhou et al. [39] observed that 39% of the evaluated D-IBS patients had increased IP, which was associated with an increased severity index score of functional bowel disorder and with hypersensitivity to visceral and thermal nociceptive pain stimuli. More recently, Li et al. [37] demonstrated that 47% of D-IBS patients with increased s-IP tend to be more severely impaired with regard to psychological effects and quality of life. In particular, the authors found that D-IBS patients with increased s-IP were experiencing higher levels of psychological stress than those with normal s-IP. It has been hypothesised that stress can lead to a more permeable intestinal wall that increases the availability of water, sodium, and energy-rich substances necessary to meet the increased metabolic demand induced by the stressors. Besides, the stress-induced increases in IP raise the possibility of bacterial translocation, which in turn can stimulate an innate and adaptive immune response [37, 40].

In the present study, the alterations of the mucosal barrier in D-IBS(+) patients resembled those found in CD patients. As for the urinary markers of permeability, D-IBS(+) showed La/Ma ratio values not significantly different from those in CD patients, but three-fold higher than those in D-IBS(−) patients. The latter group, in turn, had a La/Ma ratio equal to that of HC subjects and significantly different from that of CD patients. Of note, the two D-IBS groups did not show significant differences in Ma excretion. As a consequence, the functional integrity of Ma recovery reflecting the transcellular pathway lets us hypothesise that our D-IBS patients did not suffer from villous atrophy. Besides, per inclusion criteria, D-IBS patients had to be negative for serologic markers of CD.

Additionally, the higher La excretion in D-IBS(+) patients compared to D-IBS(−) and HC subjects suggests that impairment in the paracellular permeability characterises the small intestinal epithelium of D-IBS(+) patients. These data encourage us to further investigate the possible alterations in the TJ proteins, such as the Claudin and Occludin families [10]. Lastly, D-IBS(+) patients also had higher Su excretion than D-IBS(−) ones, with values closer to those in CD patients. This evidence allows us to hypothesise that D-IBS(+) patients might also suffer from an increase in gastro-duodenal permeability.

The urinary markers were in agreement with significantly higher levels of I-FABP and DAO observed in D-IBS(+) patients compared to D-IBS(−) ones, with the former showing values similar to those in CD patients. This evidence suggests the loss of integrity of the intestinal epithelium in D-IBS(+) patients. Enterocytes express I-FABP and DAO abundantly, and in the present study, significant correlations were found between these proteins and the La/Ma ratio in the overall population. Probably, some injury to the enterocytes could increase the release of I-FABP and DAO and compromise s-IP, even though it may not be solely responsible, as already observed in response to other physiological stressors [41].

The significant differences in these circulating proteins along with those of the La pathway proved that the D-IBS group was not a homogeneous class regarding s-IP and that two subtypes can be identified.

Moreover, the two D-IBS subtypes showed a different inflammatory status, as demonstrated by the higher IL-6, IL-8 and LPS levels in D-IBS(+) patients compared to D-IBS(−) patients. Based on these data, we can suppose that the altered GI barrier function observed in the former group may allow easier passage of bacteria and inflammatory agents through the mucous layer of the intestine. In turn, this cascade of events could also influence the course of the disease.

A major limitation of this case-control study was that the investigated parameters might not give an overview of whether bacterial translocation is mutually related to the observed alterations of s-IP in our D-IBS(+) patients. Thus, the analysis of IP in the large bowel (e.g., by analysing the urinary excretion of sucralose) as well as the investigation of the microbiota of these patients may improve the clinical relevance of the present findings. Another limitation was that the influence of hormones on gut permeability and differences between the genders concerning IBS and CD were not evaluated due to the small number of patients for gender subgroups. The role of sex steroids in the regulation of IP has not been fully elucidated, even if oestrogens can significantly modulate GI motility and visceral hypersensitivity [42]. CD is more frequent in women than men. Women suffer from nausea/vomiting and constipation, while greasy stools are more prevalent in men. Besides, depression, osteoporosis, fibromyalgia and unexplained hypochromic anaemia predominate in women [43]. Further investigation is needed to demonstrate how gender may influence s-IP as well as circulating biomarkers of GI barrier function.

## Conclusion

Our current study is of particular interest, since it demonstrates the presence of significant differences in the profiles of biomarkers related to the intestinal barrier function among HC, D-IBS, and CD patients. Besides, present data support the concept that the intestinal barrier injury and low-grade inflammation could be involved in the pathophysiology of D-IBS, even if they represent a feature that is not always detectable and two distinct D-IBS subtypes could be identified. The investigation of possible s-IP alterations (i.e., considering the La/Ma ratio) might be useful to assess better and categorise this heterogeneous D-IBS population.

## Abbreviations

%La: Percentage of ingested lactulose recovered in the urine
%Ma: Percentage of ingested mannitol recovered in the urine
%Su: Percentage of ingested sucrose recovered in the urine
BMI: Body mass index
CD: Celiac disease
C-IBS: Constipation-predominant IBS
DAO: Diamine oxidase
D-IBS: Diarrhoea-predominant IBS
ELISA: Enzyme-link immunosorbent assay
EMA: Anti-endomysium antibodies
GFD: Gluten free diet
GI: Gastrointestinal
GSRS: Gastrointestinal Symptom Rating Scale
HC: Healthy Controls
IBS: Irritable bowel syndrome
I-FABP: Intestinal fatty acid binding protein
IgA: Immunoglobulin A
IL-6: Interleukin-6
IL-8: Interleukin-8
La: Lactulose
LPS: Lipopolysaccharide
Ma: Mannitol
s-IP: Small intestinal permeability
Su: Sucrose
TJ: Tight junction
TLR-4: Toll-like receptor 4
tTG: Tissue transglutaminase
